# Effects of Physical Exercise on Executive Function in Adults with Depression: A Systematic Review and Meta-Analysis

**DOI:** 10.3390/ijerph192215270

**Published:** 2022-11-18

**Authors:** Falonn Contreras-Osorio, Rodrigo Ramirez-Campillo, Enrique Cerda-Vega, Rodrigo Campos-Jara, Cristian Martínez-Salazar, Rafael E. Reigal, Antonio Hernández-Mendo, Lara Carneiro, Christian Campos-Jara

**Affiliations:** 1 Exercise and Rehabilitation Sciences Institute, Faculty of Rehabilitation Sciences, Universidad Andres Bello, Santiago 7591538, Chile; 2 Exercise and Rehabilitation Sciences Institute, School of Physical Therapy, Department of Rehabilitation Sciences, Universidad Andres Bello, Santiago 7591538, Chile; 3 Pedagogy in Physical Education and Health Career, Department of Health Science, School of Medicine, Pontificia Universidad Católica de Chile, Santiago 7820436, Chile; 4 Psychiatry Department, Hospital Mauricio Heyermann, Angol 4650207, Chile; 5 Department of Physical Education, Sports, and Recreation, Pedagogy in Physical Education, School of Education and Social Sciences and Humanities, Universidad de La Frontera, Temuco 4780000, Chile; 6 Department of Social Psychology, Social Anthropology, Social Work and Social Services, Universidad de Málaga, 29071 Málaga, Spain; 7 Physical Education Department, College of Education, United Arab Emirates University, Abu Dhabi 112612, United Arab Emirates; 8 Research Center in Sports Sciences, Health Sciences and Human Development, CIDESD, University of Maia, ISMAI, 4475-690 Maia, Portugal

**Keywords:** executive function, inhibition, working memory, cognitive flexibility, mental processes, depressive disorder, neurocognitive disorders, exercise

## Abstract

Executive function is among the most affected cognitive dimensions in depression. Physical exercise may improve executive function (e.g., working memory, inhibition, cognitive flexibility), although this is without consensus on adults with depression. Through this systematic review, we aim to elucidate the effects of physical exercise programs on executive functions in adults with depression. The literature search was performed in four relevant electronic databases, combining keywords and medical subject headings, from inception until September 2022. Controlled interventions, involving adults with depression, and reporting working memory, inhibition, and/or cognitive flexibility pre-post-intervention data, were considered includable. Results from meta-analyses included effect size (ES, i.e., Hedges’ g) values reported with 95% confidence intervals (95%CIs), with *p* set at ≤0.05. Seven studies were included, including 202 men and 457 women (age: 21.0–51.2 years; mild–moderate depression). For working memory, a small favoring effect was observed in the experimental groups compared with controls (ES = 0.33, 95%CI = 0.04–0.61; *p* = 0.026; I^2^ = 64.9%). For inhibition, physical exercise had a small favoring non-significant effect compared with controls (ES = 0.28, 95%CI = −0.17–0.74; *p* = 0.222; I^2^ = 72.4%). Compared with the control group, physical exercise had a trivial effect on cognitive flexibility (ES = 0.09, 95%CI = −0.21–0.39; *p* = 0.554; I^2^ = 68.4%). In conclusion, physical exercise interventions may improve working memory behavioral measures in adults with mild-to-moderate depression when compared with active and passive control conditions. However, the reduced number of available high-quality studies precludes more lucid conclusions.

## 1. Introduction

According to the Global Burden of Diseases, Injuries, and Risk Factors Study, which provides a systematic scientific evaluation of published and contributed data on the incidence, prevalence, and mortality of 369 diseases and injuries for 204 countries and territories, depression is the most frequently found mental health condition among the general population, with a combined rate of 3440.1 (per 100,000) for both sexes, and being higher among women (rate, 4158.4/100,000) than in men (rate, 2713.3/100,000) [1]. This disease has become a global health issue, which not only affects people’s mental health but also increases the risk of comorbidities in comparison to the general population [2], and demands continuous effort from both families and society [3,4,5]. Based on forecasts of economic and social development worldwide, the Global Burden of Disease Project estimated that depression will be among the top three causes of disease burden by 2030 [6]. People with this condition usually exhibit loss of interest and pleasure, low self-esteem, guilt, fatigue, disturbed sleep, and cognitive symptoms that affect information processing speed, visual selective attention, verbal learning, long-term memory, and executive functioning [7,8,9,10]. Depression often has a chronic course, being in these cases associated with higher levels of depressive and somatic symptoms, along with cognitive dysfunction [8,11].

Cognitive deficit is a central feature in depression [8,9,10]. Rock et al. [9] reported that approximately two-thirds of patients with depression exhibit cognitive deficit, persisting in at least one-third of subjects in whom mood-related symptoms have already remitted. In line with this, Semkovska et al. [8] identified that deficits in selective attention, working memory, and long-term memory persist in remission from a depressive episode and worsen with repeated episodes. Thus, the cognitive deficit in depressive disorders could manifest regardless of other symptoms, although it interacts with relevant emotional and social factors to influence the individual’s social functioning [12,13,14,15].

Executive functions refer to a series of mental processes that regulate goal-directed behavior, and are responsible for the supervision and control of the mechanisms that enable the use of information [16]. They are grouped into three main dimensions as follows: working memory, inhibition, and cognitive flexibility [17,18]. These skills are among the most impacted cognitive processes and stand out because of their important contribution to the psychosocial adaptation of people with depression, even if remitted, which affects their occupational and relational performance, regardless of the clinical improvement in other types of symptoms [9,15,19,20].

Standard antidepressant therapy focuses on remission of mood-related symptoms without necessarily targeting cognitive deficits [21], which may not provide patients with sufficient tools to achieve optimal functioning in highly demanding daily tasks which place the subject in the context of a changing environment. Hence, decision-making or handling a large amount of information are essential skills as this information must be efficiently managed to meet previously set objectives (e.g., academic or work environments) [19,22,23]. In this context, physical exercise has been proposed as a low-cost and easy-to-implement therapeutic option in adults with depression in order to reduce the functional impact of cognitive symptoms, which can be used as a single therapy, adjuvant therapy, or combined therapy [19,24,25]. The physiological mechanisms that have been described to mediate this effect include the expression of neurotransmitters and neurotrophic factors (e.g., Brain-Derived Neurotrophic Factor), synaptic plasticity, the modification of inflammatory pathways, and the activation of hormone-dependent mechanisms and the cerebrovascular function [26,27,28,29,30].

Physical exercise uses specific movement patterns performed in a systematic manner through a program planned to improve fitness or health-related outcomes [31]. Clinical research assessing the effects of chronic exercise on cognition in adults with depression has predominantly used aerobic exercise training programs [32,33,34,35], although it has also considered strength training [36] or multicomponent training (coordination, endurance, and strength training) [30]. The results obtained in these investigations in adults with depression show improvements in short-term memory [34,35], inhibitory control [33], processing speed [35,37], attention, verbal fluency, and cognitive flexibility [37] after the implementation of sustained exercise programs for weeks and months. However, other studies have not shown improvements in cognitive measures when comparing physical exercise with the control condition in this population [36,38]. Hoffmann et al. [38] state that the lack of effect of their intervention program (aerobic exercise versus placebo) on the cognitive functioning of adults with depression could be explained by the absence of baseline cognitive impairment among participants, the characteristics of the diagnosis of depression (nonrecurring, early onset mild-to-moderate depression with good response to treatment), the relatively short duration of treatment (4 months in both studies), and a small percentage of improvement in the aerobic capacity of participants (6%), which could have been insufficient to elicit effects on the cognitive functioning at the end of the intervention. However, Krogh et al. [36] suggest that the absence of significant effects of their four-month training programs (strength training versus relaxation and aerobic training versus relaxation) on the cognitive abilities of adults with depression could be due to a possible antidepressant effect of the control condition, non-blinded treatment allocation for patients and therapists, inclusion of participants who had received previous pharmacological treatment for more than 6 weeks, low attendance at training programs (approximately 50%), low weekly scheduled frequency (twice a week), and the possible absence of baseline cognitive impairment of patients.

Other systematic reviews have focused on the effects of exercise on depressive mood-related symptoms [24,39,40,41,42], although to date, only two previous reviews and meta-analyses have assessed the impact of physical exercise on cognitive symptoms in adults with depression [22,43], including global cognition and different cognitive domains as outcome measures (e.g., processing speed, attention/vigilance, verbal learning and memory, and visual learning and memory). Supplement The cognitive domains selected in both studies were categorized according to the structure of the MATRICS Consensus Cognitive Battery (MCCB) [44]. This makes it possible to compare the results obtained between both reviews, although it does not allow for the visualization of the results with respect to the three main dimensions of executive function (namely, working memory, inhibition, and cognitive flexibility), which are especially relevant in this population’s symptomatology and functional performance [45,46,47,48]. In addition, the previously mentioned reviews [22,43] included intervention studies with a meditation component [49] in their meta-analyses, which limits the interpretation of their results regarding the effect of physical exercise. Moreover, the increasing number of publications in this field will likely render any systematic review quickly outdated. Indeed, it has been shown that in rapidly emerging research fields, 25% of systematic reviews become obsolete within 2 years and 50% within 5 years [50]. Considering that the aforementioned reviews [22,43] were conducted in 2017, an update on the topic would be advisable.

Therefore, we aim to synthesize the available scientific articles related to the effects of physical exercise programs on executive functions in adults with depression, through a systematic review and meta-analysis. This research approach may provide a deeper insight on the currently available scientific literature related to the effects of physical exercise as a potential non-pharmacology strategy to contribute to the treatment of adults with depression. Our research question was: what are the effects of physical exercise interventions, compared with an active or passive control condition, on the executive functions (working memory, inhibition, and cognitive flexibility) in adults with depression?

## 2. Materials and Methods

### 2.1. Search Strategy

**Figure 1:**
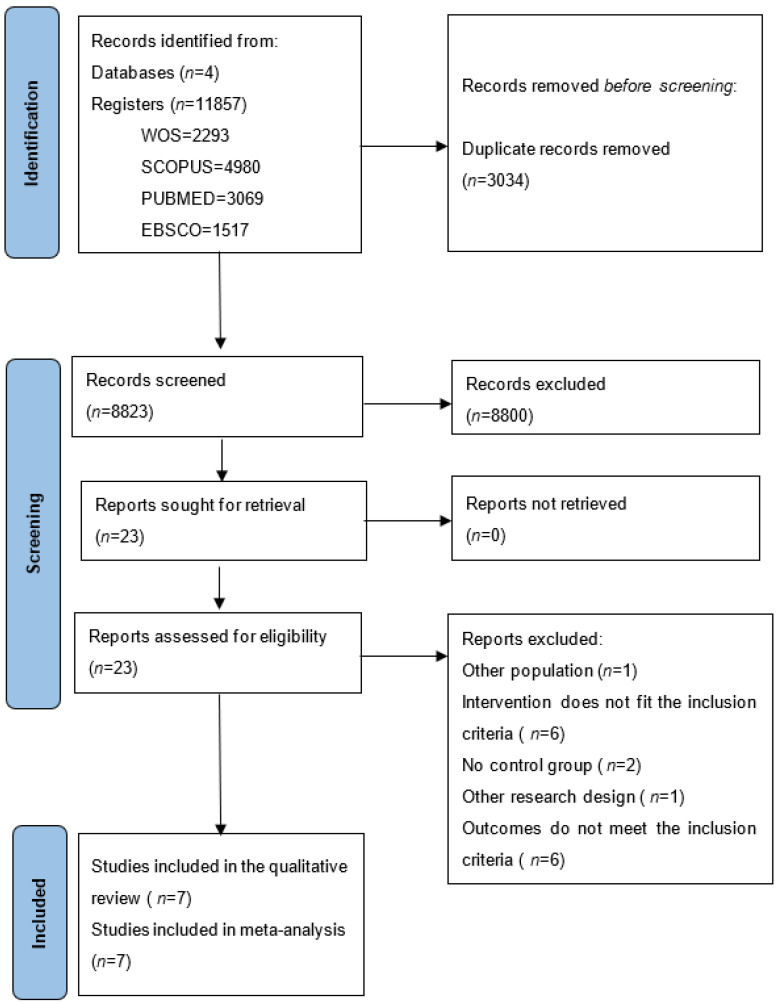
A flowchart of the studies included in the meta-analysis.

This protocol was prepared following the guidelines established by Preferred Reporting Items for Systematic reviews and Meta-Analysis Protocols (PRISMA-P) [51] (Figure 1). We pre-registered our meta-analytic review in the International Prospective Register of Systematic Reviews (CRD42022358339).

The electronic databases Web of Science, PubMed, Scopus, and EBSCO were searched from inception to September 2022, without filters or limits (e.g., language). The search strategy used in each database included a combination of different medical subject headings (MeSH) or synonyms aimed at identifying and assessing relevant studies. Terms used for concept 1 included “executive function”, “cognitive function”, “cognition”, “inhibitory control”, “inhibition”, “working memory”, “executive functioning”, and “cognitive flexibility”. Terms used in concept 2 were “sport”, “modified sport”, “fitness”, “exercise”, “physical activity”, “athletics”, and “sport practice”. Terms used in concept 3 were “depression”, “depressive disorder”, “mood disorders”, and “major depressive disorder” (Appendix A. Table A1. Specific Search Strategy for Each Database).

As a supplement to the aforementioned procedure, a manual search was conducted in the reference lists of previous related reviews and all studies included in order to identify other potentially eligible trials. Systematic reviews were searched in the same databases using the filters “systematic review” or “review” after the usual search strategy. Two external experts in executive functions were consulted to check the list of articles included and to identify possible additional articles that could be included in the list. The experts were included based on the Expertscape rank for “Executive + function” that can be found through the link: https://www.expertscape.com/ex/executive+function (accessed on 21 September 2022).

### 2.2. Eligibility Criteria

The eligibility criteria were defined based on participants (P), interventions (I), comparators (C), outcomes (O), and study (S) design (PICOS) (Table 1). The PICOS approach allows clear formulation of research questions related to clinical problems, and facilitates the development of literature search strategies, including key elements for the synthesis of the currently available research evidence on a particular topic [52], allowing a better transference of results to clinical practice.

Studies published as original articles in peer-reviewed journals were selected. Studies were available in full text and contained sufficient data to calculate the effect size (ES).

Eligible studies were required to report pre–post-intervention data (e.g., mean; standard deviation) for one or more direct assessment measures of executive functions (working memory, inhibition, or cognitive flexibility). These tasks were required to be applied to the participants through validated instruments for adults. Some examples of tasks to measure working memory, inhibition, and cognitive flexibility are the N-back task [53], Stroop task [54] and Trail Making Test—Part B [55], respectively. Indirect or global measures of executive functions were excluded, since they cannot be compared with the cognitive processes included.

According to previous studies, a minimum effective duration of 3 weeks was determined for the intervention programs [30,35]. The definition of exercise used corresponds to “a type of physical activity consisting of planned, structured, and repetitive bodily movement, aimed at improving and/or maintaining one or multiple components of physical fitness” [56].

### 2.3. Data Management

The articles were imported into a reference management system, where duplicates were removed. Subsequently, two independent authors (FCO and CCJ) examined the titles and abstracts of the articles found in the databases, applying the same inclusion criteria to determine compliance with said conditions by following the PICOS method (see Supplementary Data: search strategy and criteria inclusion). Discrepancies were resolved with a third author (RRC) until a consensus was reached.

The reference lists of articles included and reviews retrieved from the original database search were examined. A PRISMA flowchart [51] was used to document the selection process, as well as the corresponding exclusion criteria.

### 2.4. Data Extraction

One reviewer (FCO) completed the data extraction independently, which was verified by a second reviewer (CCJ). Data extracted from the studies selected were: author, year of publication, sample size, characteristics of the participants (sex, age, fitness level, psychiatric diagnosis, severity, comorbidities, and pharmacological treatment), description of the exercise training program, intervention length (i.e., weeks), weekly frequency, session length (i.e., minutes) and intensity, control condition, dimensions of the executive function assessed (i.e., inhibition, working memory, or cognitive flexibility), and tasks used (for example, Trail Making Test—Part B to evaluate cognitive flexibility).

The means and standard deviation values of the dependent variables were extracted before and after the exercise interventions of the studies included using Microsoft Excel (Microsoft Corporation, Redmond, WA, USA). Two authors (FCO and CCJ) extracted data independently, and any discrepancies between the authors (e.g., mean value for a given outcome, number of participants in a group) were resolved by consensus with a third author (RRC) [57].

### 2.5. Risk of Bias (Quality) Assessment

The methodological quality of the studies included was assessed using the Physiotherapy Evidence Database (PEDro) scale, as had been accomplished previously [57,58,59,60]. The methodological quality of the studies was construed using the following convention [61,62,63]: ≤3 points “poor” quality, 4–5 points “moderate” quality, and 6–10 points “high” quality. For trials previously rated in the PEDro database, their corresponding score was adopted. The methodological quality of the studies included was independently reviewed by two reviewers—FCO and CCJ—and any discrepancies between them were resolved by consensus with a third reviewer (RRC).

All studies meeting the inclusion criteria were incorporated into the review, regardless of their methodological quality.

### 2.6. Strategy for Data Synthesis

The meta-analyses were performed following previous instructions [57]. In short, meta-analysis was only conducted when ≥3 studies were available [64,65]. Effect sizes (ESs, i.e., Hedges’ g; with 95% confidence intervals [95%CIs]) were calculated for the main outcomes using the DerSimonian and Laird random effects model, and interpreted as: <0.2 trivial, 0.2–0.6 small, >0.6–1.2 moderate, >1.2–2.0 large, >2.0–4.0 very large, and >4.0 extremely large [66]. Heterogeneity was assessed with the I^2^ statistic and categorized as low (<25%), moderate (25%–75%), and high (>75%) [67]. All analyses were performed using the Comprehensive Meta-Analysis software (version 2, Biostat, Englewood, NJ, USA). Statistical significance was established at *p* ≤ 0.05.

## 3. Results

### 3.1. Study Selection

The electronic search process identified 11,857 studies (2293 from WOS; 4980 from SCOPUS; 3069 from PUBMED; and 1517 from EBSCO). Duplicate studies were removed (*n* = 3034), the titles and abstracts of the remaining studies were reviewed, and another 8800 documents were removed. Next, the full text versions of 23 studies were reviewed, and 16 of them were rejected because they considered a different type of population (*n* = 1), the intervention did not meet the inclusion criteria (*n* = 6), they did not include a control group (*n* = 2), they considered a different research design (*n* = 1), or they assessed other cognitive skills (*n* = 6). This systematic review and meta-analysis included the seven remaining studies [30,33,34,35,36,37,38], in which working memory was analyzed in six studies [30,34,35,36,37,38], inhibition in four [30,33,34,38], and cognitive flexibility in six [30,34,35,36,37,38]. The studies included considered eight experimental groups that corresponded to 386 participants, and seven control groups included 273 participants, with a chronological age that ranged from 21.0 to 51.2 years. Table 2 shows the details of the participants’ characteristics.

### 3.2. Study Characteristics

The interventions mostly consisted of aerobic exercise programs, although one of the studies introduced a strength training program [36] and another one involved a physical activity program including coordination, endurance, and strength exercises [30]. The interventions were implemented in addition to routine pharmacological treatments for depression [30,33,34,35,36,37], with the exception of Hoffman et al.,[38] where none of the experimental group participants received pharmacological therapy during the intervention period. One of the studies [36] included psychotherapy as part of the treatment for participants in its experimental and control groups. The total duration of the programs ranged from 3 weeks [30] to 4 months [36,38], with 2–3 weekly sessions and 30–90 min per session. The control conditions reported were active and passive, considering placebo pills [38], relaxation training [36], stretching exercise [33,34], routine treatment (drug therapy) [37], cooperative group games with other participants [30], and occupational therapy [35]. Table 3 shows a detailed description of these conditions and other aspects associated with the intervention programs.

### 3.3. Methodological Quality

Table 4 shows the results of the methodological quality assessment using the PEDro scale for the seven studies included. In total, four studies received 4–5 points and were classified as “moderate” for quality, whereas three studies received 6–10 points and were thus classified as “high” for methodological quality.

### 3.4. Meta-Analysis Results of the Effects of Interventions on Working Memory

**Figure 2:**
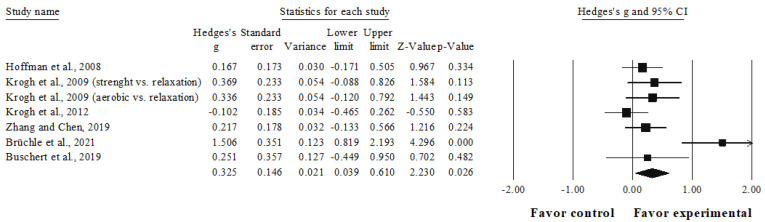
Forest plot for working memory outcome. Values shown are effect sizes (Hedges’s g) with 95% confidence intervals. The size of the plotted squares reflects the statistical weight of the study. Black squares: individual studies. Black rhomboid: summary result.

The meta-analysis for working memory involved six studies, seven experimental groups (*n* = 371), and six control groups (*n* = 258). Compared with the control group, a small favoring significant effect was observed in the intervention group (ES = 0.33, 95%CI = 0.04–0.61; *p* = 0.026; I^2^ = 64.9%; Figure 2).

### 3.5. Meta-Analysis Results of the Effects of Interventions on Inhibition

**Figure 3:**
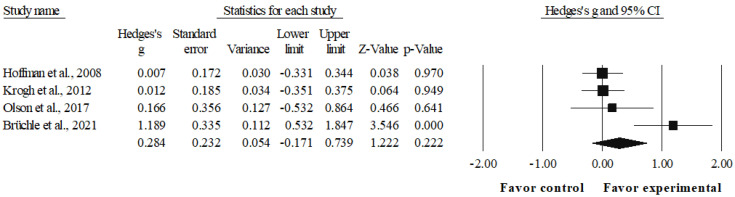
Forest plot for inhibition outcome. Values shown are effect sizes (Hedges’s g) with 95% confidence intervals. The size of the plotted squares reflects the statistical weight of the study. Black squares: individual studies. Black rhomboid: summary result.

The meta-analysis for inhibition involved four studies, four experimental groups (*n* = 198), and four control groups (*n* = 141). Compared with the control group, a small favoring non-significant effect was observed in the intervention group (ES = 0.28, 95%CI = −0.17–0.74; *p* = 0.222; I^2^ = 72.4%; Figure 3).

### 3.6. Meta-Analysis Results of the Effects of Interventions on Cognitive Flexibility

**Figure 4:**
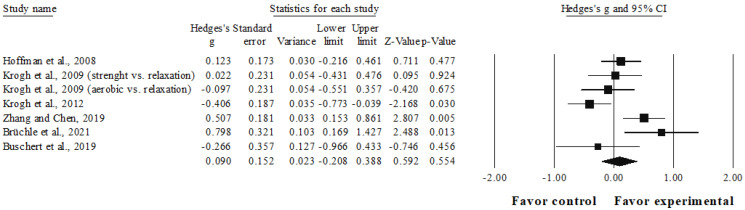
Forest plot for cognitive flexibility outcome. Values shown are effect sizes (Hedges’s g) with 95% confidence intervals. The size of the plotted squares reflects the statistical weight of the study. Black squares: individual studies. Black rhomboid: summary result.

The meta-analysis for cognitive flexibility involved six studies, seven experimental groups (*n* = 371), and six control groups (*n* = 258). Compared to the control group, a trivial effect was observed in the intervention group (ES = 0.09, 95%CI = −0.21–0.39; *p* = 0.554; I^2^ = 68.4%; Figure 4).

### 3.7. Adverse Effects

None of the studies included reported assessing or reporting data regarding adverse effects resulting from the exercise interventions (e.g., soreness, pain, fatigue, or injury).

## 4. Discussion

The studies available reveal that physical exercise interventions have a small but significant favorable effect on working memory in adults with mild-to-moderate depression compared with the control groups. The effect on inhibition capacity was also small, although not significant, whereas only a non-significant trivial effect on cognitive flexibility could be observed.

### 4.1. Working Memory

Working memory is an essential cognitive system needed for daily living activities as it allows for the temporary storage, manipulation, and updating of relevant information during the performance of complex tasks in different contexts [68,69]. The outcomes of this meta-analysis indicate that physical exercise training improves working memory in adults with depression, with a small but significant positive effect (ES = 0.33, *p* = 0.026) in the intervention group compared with the control group.

This is contrary to what had been reported in previous meta-analyses [22,43], where no evidence of the significant effects of physical exercise interventions on working memory could be observed in adults with depression. These systematic reviews included only three of the studies considered in this research [34,36,38] since Zhang and Chen [37], Brüchle et al. [30], and Buschert et al. [35] were published at a later time than previous systematic reviews. This update of the studies selected in the working memory outcome could have contributed to the positive results obtained because of the influence of their exercise programs, especially the study by Brüchle et al. [30], which revealed the largest effect size within the working memory dimension (ES = 1.51). In said research paper [30], only results from participants who completed all intervention sessions were reported, and improvements in cognitive symptoms were accompanied by a significant increase in neuroplasticity after the moderate-intensity exercise program, particularly in participants with higher psychological/affective severity symptoms at the beginning of the intervention program.

Previous research has highlighted the likelihood that final cognitive outcomes could be related to the baseline severity level of psychological/affective symptoms in patients with depression receiving a physical exercise intervention [37,38,43]. In this systematic review, participants in the selected studies were rated at baseline as having mild-to-moderate-severity depression using scales such as the Hamilton Depression Rating Scale or the Beck Depression Inventory, 2nd edition. This could indicate little potential for improvement considering that greater severity of depression has been associated with lower cognitive performance [45], and that cognitive improvement could be greater in patients with cognitive impairment compared with those participants whose cognitive level lies within normal ranges [43,70,71]. In the working memory dimension, only one study reported decreased cognitive performance among its participants [37], two studies reported no cognitive impairment [30,38], and three studies provided no information in this regard [34,35,36].

Hoffman et al. [38] provide additional background when comparing the pre–post-intervention performance in working memory of the upper tertile of their sample with respect to the average change in maximum oxygen consumption (*n* = 27; average change in peak VO_2_ = 15.60%), evidencing a significant effect compared with that of participants in the control group (*p* = 0.04). These results are consistent with those of the study conducted by Greer et al. [71], where significant improvements in working memory could be observed after an aerobic exercise training program in adults with depression. Falkai et al. [72] suggest that exercise interventions intended for people with major depression should aim at increasing cardiorespiratory fitness, based on the recommendations by the American College of Sports Medicine (150 min moderate-intensity training per week or vigorous-intensity exercise training for 75 min per week) in order to achieve improvements in the cognition and global functioning of these patients. Said recommendation [73] was only fulfilled in the study by Brüchle et al. [30] by all reported participants, being the only study included in this meta-analysis to evidence significant outcomes in favor of the experimental group in all its executive function measures (working memory, inhibition, and cognitive flexibility). Conversely, although Krogh et al. [36] planned their interventions following this indication, the low level of compliance showed by their participants regarding the intervention plans (56.2% in the strength group and 50.6% in the aerobic group) could have explained the lack of effect obtained in their experimental groups regarding the control conditions for the dimensions assessed (working memory and cognitive flexibility). Other studies included in this review [33,34,35,38] considered lower doses than those suggested by Falkai et al. [72]. Zhang and Chen [37] did not report enough information to perform this analysis.

### 4.2. Inhibition

“Inhibition” refers to the ability to suppress internal or external stimuli that interfere with the ability to choose an appropriate response to the purpose of the task [74]. These results add to the currently existing body of evidence given that this executive function dimension has not been analyzed in previous meta-analyses in adults with depression. The outcomes of the present meta-analysis revealed that the intervention had a small non-significant favoring effect compared with the control group (ES = 0.28, *p* = 0.222). Despite the lack of global significance in this dimension, the study by Brüchle et al. [30] stands out because they saw a significant positive effect from their intervention program (ES = 1.18). These findings are consistent with previous studies on healthy adults, where a higher level of physical activity is associated with better executive functions throughout life [75,76]. However, the other experimental groups [33,34,38] did not show significant improvements in inhibition compared with the control conditions assessed. Hoffman et al. [38] analyzed the clinical characteristics of their participants (for example, mild-to-moderate severity, nonrecurring depression, and good response to treatment) as a possible explanation for their results, whereas Krogh et al. [34] stated that low attendance may have been decisive for the lack of effect of their intervention program on depressive and cognitive symptoms (with the exception of visuospatial memory performance, which did show a significant improvement after the intervention program). Previous studies have highlighted the relationship between adherence to treatment and improvement in cognitive performance, suggesting that low attendance at exercise sessions could be related to a lower effect of intervention programs on the cognitive performance of participants as the minimum threshold necessary to observe that improvement is not reached [22,43,71,77,78].

Monteiro et al. [77] assessed the clinical and demographic factors related to adherence to exercise programs by patients with depression. According to this study, although adults with mild depressive symptoms were more likely to be physically active, smoking, being divorced, having melancholic symptoms (according to Mini-International Neuropsychiatric Interview), a lower global functioning (using Global Assessment Functioning), and lower scores in the psychological domain of quality of life (using the World Health Organization Quality of Life Questionnaire—Brief Version) were associated with less participation in exercise programs. Considering these variables in future research could help differentiate patients at higher risk of dropout from those who might have better adherence to physical exercise interventions. This would allow the targeting of support strategies aimed at improving the level of motivation and adherence to physical exercise interventions of patients with depression who present a higher risk of desertion [77,79].

However, Olson et al. [33] used a behavioral measure (response accuracy and reaction time) and event-related potentials (ERP) (i.e., N2 amplitude) as a direct measure of neural transmission in a flanker task to assess inhibitory control. In this meta-analysis, only the behavioral measure was included as it allowed for comparison with the measures used in the other studies, observing a non-significant effect in favor of the experimental group. However, this research study identified significant improvements in inhibition by the change in the amplitude of the N2 ERP component after the intervention, compared with its control group. These results could be explained by the different capacities of the measures used to detect cognitive changes [80], the size of the sample used (*n* = 30), or the relatively short duration (8 weeks) of the intervention program [33].

### 4.3. Cognitive Flexibility

Cognitive flexibility makes it possible to respond to the demands of the environment by changing the focus of attention or by searching for alternative ways of solving tasks that entail new priorities [81]. As for inhibition, this dimension of the executive function has also not been assessed in previous meta-analyses in adults with depression. The results of this meta-analysis showed that the intervention had a trivial effect when compared with that of the control group (ES = 0.09, *p* = 0.554). However, among the studies included in this dimension, two of them revealed significant results in favor of the experimental group [30,37].

The results obtained in the three dimensions of the executive functions assessed must be analyzed with caution since the number of studies included is limited and there are several factors that have not yet been resolved with regard to the research design in this area, such as the most appropriate type of control group to be used in studies on exercise and depression, the influence of participants’ baseline cognitive level, and the development of exercise programs that address the motivation of participants with depression, which constitutes a crucial variable to ensure a level of adherence that would allow more solid conclusions to be drawn [24,43,72]. Attention should also be paid to the qualitative characteristics of the exercise [82], as well as to the control condition characteristics. In this regard, Ranjbar et al. [78] recommended the waiting list as a control condition to be compared with the experimental group in this type of study in people with depression. However, Hu et al. [83] suggested that the different control conditions (e.g., psychosocial interventions, waiting list, no treatment) used in studies analyzing the effect of physical exercise interventions on depression may represent an important source of heterogeneity, thus contributing to the differences found in the effect sizes.

Regarding the methodological quality of the included studies, none of them exceeded six points regarding the aspects with less compliance, the blinding of subjects and therapists, the attainment of measurements above the 85% of the subjects initially assigned to the groups, and the concealment of group allocation from subjects. These are critical factors that could be improved in future research. Previous reviews by Sun et al. [22] and Brondino et al. [43] agree on this aspect, since none of the studies included in their investigations complied with a proper blinding of the participants or the personnel in charge of executing the intervention programs, in addition to evidencing other limitations such as the drop-out bias and the inadequate allocation concealment.

### 4.4. Potential Limitations and Suggestions for Future Research

Some limitations need to be taken into account when interpreting the results. (1) The results of our systematic review evidenced the existence of few controlled studies with sufficient methodological quality, meaning that the results exhibited the need to be interpreted with caution. (2) Some of the studies included did not report relevant information such as the baseline cognitive level (*n* = 4) or the comorbidity of their participants (*n* = 2). (3) There is great diversity regarding the way in which studies report compliance with their intervention programs, which hinders their comparison. Future research should detail the baseline characteristics of participants, especially with respect to the previously mentioned aspects, in addition to reporting the level of compliance with their intervention programs in a more homogeneous way. (4) The small number of studies included in this review limits the analysis of possible moderators impacting the results obtained. (5) None of the studies selected included participants with severe depression; hence, the interpretation of the results obtained could be extrapolated only to patients with mild-to-moderate depression. Future studies should focus on investigating the effect of physical exercise (aerobic, strength, coordination, or multicomponent training) on the executive functions of subjects with cognitive deficits exhibiting different levels of severity of depression, and that implement strategies aimed at promoting motivation as a way to improve adherence to treatment throughout the intervention period and that adapt to the various real clinical contexts in which the treatments of these subjects are implemented. (6) Although none of the studies included reported adverse events related to exercise programs, it remains unclear whether a detailed record of these data was actually made. Recommendations for future studies include the monitoring and description of possible injuries, pain, or any other potential adverse effects that could contribute to understanding the safety of physical exercise programs in adults with depression.

Future studies may assess the effects of physical exercise on healthy life styles in participants with depression, in addition to the symptoms associated with depression. This may help to induce more long-term beneficial effects. Additionally, future research may assess the impact of group-based physical exercise interventions, with a greater potential to reduce isolation and improve self-esteem and social cognition [84,85].

## 5. Conclusions

Physical exercise interventions may improve behavioral measures of working memory in adults with mild-to-moderate depression. This improvement can be observed when comparing physical exercise with passive (e.g., placebo pill) and active control conditions (e.g., cooperative group games). These conclusions result from the analysis of seven studies of moderate-to-high methodological quality, with a moderate impact of heterogeneity (I^2^). Future studies should provide further clarification on the effect of exercise interventions on cognitive inhibition and flexibility, thus contributing to increasing the existing body of evidence in order to offer effective therapeutic alternatives that allow for the improvement of the daily functioning of this population, especially when facing contexts requiring efficient information management.

Future studies are encouraged to include more detailed characteristics regarding the physical exercise interventions (e.g., compliance; attrition; potential adverse effects; motivation strategies), and participants’ characteristics (e.g., social background; sport history).

## Figures and Tables

**Table 1:** Eligibility Criteria Based on PICOS.

PICOS	Inclusion Criteria	Exclusion Criteria
1. Population	1.1. Adults (age, 18–65 years) diagnosed with major depression or unipolar depression according to the criteria of a validated instrument, such as the Diagnostic and Statistical Manual of Mental Disorders, Fourth Edition (DSM-IV), or the International Classification of Diseases 10th Revision (ICD-10), without restrictions based on sex or fitness level.	1.1. Children, adolescents, or older adults. 1.2. Presence of another primary neurological or psychiatric diagnosis, such as dementia, bipolar disorder, or psychosis. 1.3. Medical comorbidities that limit participation in physical exercise activities (e.g., significant musculoskeletal difficulties). 1.4. Active drug or alcohol abuse or dependence. 1.5. Pregnant or lactating women. 1.6. Intellectual disability.
2. Intervention	2.1. Chronic exercise intervention programs (with a minimum duration of 3 weeks) as independent therapy or complementary to usual psychiatric treatment (e.g., pharmacological treatment). 2.2. The interventions should involve aerobic training or strength training or programs combining different types of exercises (e.g., coordination, endurance, or strength training).	2.1. Acute interventions. 2.2. Chronic exercise intervention programs combined with meditation. 2.3. Chronic interventions that are not related to physical exercise.
3. Comparator	3.1. A group made up of adults diagnosed with depression not exposed to physical exercise intervention. 3.2. The control condition may be active (e.g., relaxation techniques) or passive (e.g., placebo).	3.1. Absence of control group.
4. Outcome	4.1. Pre–post-intervention values for one or more direct assessment measures for the executive functions of working memory, inhibition, or cognitive flexibility.	4.1. Indirect measures of executive functions (e.g., questionnaire).
5. Study design	5.1. Longitudinal studies with at least one experimental group and one control group that include pre- and post-intervention measurements.	5.1. Cross-sectional studies.

**Table 2:** Subjects’ characteristics from the studies included.

References	N	Sex (M/F)	Age (Years)	Diagnosis	Diagnostic Instruments	Baseline Depressive Symptom Severity	Baseline Cognitive Status	Comorbidity
Hoffman et al., 2008 [38]	153; EG: 104, CG: 49	37/116	EG: 51 ± 7, CG: 51.2 ± 7.8	Major depressive disorder	DSM-IV-TR criteria; BDI-II (≥12); HAM-D	HAM-D: 16.8 ± 4.3 (mild-to-moderate)	No cognitive impairment	Hypertension EG: 27, CG: 14; diabetes EG: 7, CG: 4; smoking EG: 15, CG: 8; alcohol (≥3 alcoholic drinks per week) EG: 7, CG: 5
Krogh et al., 2009 [36]	165; EG1: 55, EG2: 55, CG: 55	43/122	EG1: 41.9 ± 8.7, EG2: 38.1 ± 9.0, CG: 36.7 ± 8.7	Unipolar depression	ICD-10 and DSM-IV criteria; Major Depression Inventory	HAM-D-17: 17.8 ± 3.8 (mild-to-moderate)	Not reported	Not reported
Krogh et al., 2012 [34]	115; EG: 56, CG: 59	38/77	EG: 39.7 ± 11.3, CG: 43.4 ± 11.2	Major depression	DSM-IV criteria; Danish version of the Mini-International Neuropsychiatric Interview; HAM-D17 (>12)	HAM-D-17: 18.9 (95%CI 13–28) (moderate)	Not reported	Generalized anxiety EG: 33, CG: 34; Hypertension EG: 11, CG: 12
Olson et al., 2017 [33]	30; EG: 15, CG; 15	6/24	EG: 21.0 ± 1.9, CG; 21.2 ± 2.2	Major depressive disorder	ICD-10, DSM-IV and DSM-V criteria; MINI	BDI-II EG: 24.5 ± 11.5, CG: 24.3 ± 11.9 (moderate)	Not reported	Anxiety (~18%)
Zhang and Chen, 2019 [37]	125; EG: 63, CG: 62	44/81	EG: 31.4 ± 7.2, CG: 32.2 ± 7.6	Single phase depression	DSM-IV criteria	HAM-D EG: 10.3 ± 3.4, CG: 10.0 ± 3.8 (mild)	Decreased processing speed, attention, memory, verbal fluency and executive function (at least 1 SD lower than the standardized value)	Hypertension, diabetes
Brüchle et al., 2021 [30]	41; EG: 23, CG: 18	23/18	EG: 33.3 ± 3.06, CG: 40.11 ± 3.63	Major depressive disorder	ICD-10 criteria; BDI-II ≥ 10; HAM-D-17 ≥ 9	HAM-D-17 EG: 19.17 ± 0.78, CG: 17.83 ± 0.75; BDI-II EG: 27.74 ± 1.44, CG: 26.11 ± 1.77 (moderate)	No cognitive impairment	Not reported
Buschert et al., 2019 [35]	30; EG: 15, CG: 15	11/19	EG: 47.27 ± 6.84, CG:47.47 ± 8.47	Unipolar depression	ICD-10 criteria	BDI-II EG: 22.40 ± 8.53, CG: 18.27 ± 11.56; HAM-D-7 EG: 11.00 ± 3.42, CG: 9.67 ± 4.21 (moderate)	Not reported	Other mood disorders (EG: *n* = 1), neurotic, stress-related and somatoform disorders (EG: *n* = 1; CG *n* = 3), and disorders of adult personality and behavior (EG: *n* = 1; CG: *n* = 1)

BDI-II: Beck Depression Inventory, 2nd edition; CG: control group; CI: confidence interval; DSM-IV: Diagnostic and Statistical Manual of Mental Disorders, 4th ed.; DSM-IV-TR: Diagnostic and Statistical Manual of Mental Disorders, IV: Text Revision. 4th ed.; DSM-V: Diagnostic and Statistical Manual of Mental Disorders, 5th ed.; EG: experimental group; F: female; HAM-D: Hamilton Depression Rating Scale; HAM-D-17: 17-item Hamilton Depression Rating Scale; HAM-D-7: Toronto short version of the Hamilton Depression Scale; ICD-10: International Classification of Diseases, 10th Revision; M: male; MINI: Mini Neuropsychiatric Diagnostic Interview; N: number of subjects; SD: standardized deviation.

**Table 3:** Intervention characteristics of the studies included.

References	Exercise Program	Control Condition	Compliance with the Intervention Program	Length of Intervention	Weekly Frequency	Length of Sessions	Intensity	Other Treatments	Executive Function Tasks
Hoffman et al., 2008 [38]	Supervised exercise: (1) 10 min walking warm-up exercise, (2) 30 min walking or jogging on a treadmill at an intensity that would maintain their heart rate within the assigned training range, (3) 5 min cool-down exercises. Home-based exercise: Participants in a home-based exercise program received the same exercise prescription. Telephone follow-up and follow-up through scheduled visits during the intervention period.	Placebo pill	68% of home exercisers completed at least 75% of the 48 scheduled sessions compared with 67% of supervised exercisers. Treatment completers attended an average of 38.8 exercise sessions.	16 weeks	3/week	45 min	70%–85% of HRR.	CG: for insomnia, use of a hypnotic (zolpidem) no more than four doses during treatment.	Animal naming, COWAT, digit symbol subtest (WAIS-R), digits backward, Ruff 2 & 7 test, Stroop color and word test, TMT B-A.
Krogh et al., 2009 [36]	Strength group: Circuit-training program with six exercises on machines involving large muscle groups: leg extension, leg press, total abdominal, lower back, chest press, and vertical traction. As a supplement to this, free weights and sandbags were used for exercising the calf muscles, the arm abductors, the triceps muscles, and the hip abductors. Aerobic group: 10 different aerobic exercises using large muscle groups. Machines were used for cycling, running, stepping, abdominal exercises, and rowing. Additional exercises were sliding movements on small carpets, trampoline, step bench, jump rope, and Ski Fitter.	Relaxation training	The mean participation was strength group: 18.0 (56.2%), aerobic group: 16.2 (50.6%), and control group: 10.5 (32.8%) sessions of the 32 sessions.	4 months	2/week	90 min	Strength group: 50%–75% of RM. Aerobic group: 70%–89% of HRmax. Relaxation group: <12 on the Borg scale.	Antidepressant medication EG1: 39, EG2: 37, CG: 38; psychotherapy EG1: 24, EG2: 28, CG: 25	Digit span test, subtracting serial sevens, digit symbol test, verbal fluency S and animals, TMT—Part B.
Krogh et al., 2012 [34]	Aerobic training: (1) 10 min general low-intensity warm-up, (2) 30 min aerobic exercise on a stationary cycle ergometer, (3) 5 min low-intensity cool-down period.	Stretching exercises	Average attendance: 36%. Mean attendance EG: 13.5 sessions (range 0–34 and SD 9.4), CG: 12.5 sessions (range 0–34 and SD 9.3) of a planned total of 36 sessions.	3 months	3/week	45 min	65%–80% of HRmax.	Antidepressant medication EG: 4, CG: 8	Digits backward, subtracting serial sevens, Stroop test, TMT—Part B, digit symbol subtest (WAIS-R), verbal fluency S and animals.
Olson et al., 2017 [33]	45 min of continuous steady-state exercise performed on a treadmill or cycle ergometer at a prescribed moderate intensity.	Stretching exercises	75% retention rate.	8 weeks	3/week	30–45 min	40%–65% of HRR.	Antidepressant medication (∼14%).	Modified flanker task.
Zhang and Chen, 2019 [37]	Participants were asked to jog 3 km each time.	Routine treatment (drug therapy)	Four cases were lost in the experimental group.	8 weeks	3/week	Not defined	Target rate: 170-age. If the patient had high blood pressure, diabetes or other physical body disease, a heart rate of 110 bpm was indicated.	Routine treatment (drug therapy)	Wechsler Memory Scale Revised in China digit span test (backward), VF test, TMT-B.
Brüchle et al., 2021 [30]	Exercise program: Each session lasted 60 min (without breaks) and focused on one of the three exercise types once a week, namely, coordination, endurance, or strength training. These three exercise sessions were repeated every week. Each session began with a 10 min warm-up which combined physical and cognitive tasks, by coding certain movements (e.g., circling of arms, lifting knees up) with colors (colored cards held up by the instructor). The color-movement associations changed randomly in every session.	Cooperation group games with other participants.	Out of 50 recruited patients, 23 (of 25) in the experimental group and 18 (of 25) in the control group completed the study. Only results of patients who participated in all intervention sessions during the 3-week intervention period are reported.	3 weeks	3/week	60 min	Mean heart rate EG: 126.84 ± 2.83 bpm, CG: 83.02 ± 3.08 bpm.	Medication (e.g., with antidepressants) was not changed during the intervention period. Benzodiazepine <1 mg/day lorazepam equivalent. Excluded: anticonvulsive medication, lithium, and antipsychotics.	Work performance series, N-back verbal test (2-back), TMT-B, response inhibition task, Stroop interference test.
Buschert et al., 2019 [35]	Endurance training: The sessions comprised outdoor walking, Nordic walking, or running in groups of up to five patients. In adverse weather conditions, patients exercised indoors on stationary bicycles.	Occupational therapy in a group	Overall dropout rate: 21%. Number of treatment sessions received in this study EG: mean = 10.00 (SD = 2.95); CG: mean = 14.27 (SD = 8.18).	3–4 weeks	2–3/week	30 min	85% of HRmax, calculated as 200 minus age in years was taken as the upper limit.	Groups did not differ over the course of the entire treatment regarding psychopharmacological treatment (no antidepressive mediation, tri-/tetracylics, selective antidepressants, or changes in the medication).	Digit span backward, computer-assisted cardsorting procedure (Computergestütztes Kartensortierverfahren, CKV).

bpm: beats per minute; CG: control group; COWAT: Controlled Oral Word Association Test; EG: experimental group; HR: heart rate; HRmax: maximum heart rate; HRR: heart rate reserve; RM: repetition maximum; SD: standard deviation; TMT: Trail Making Test; VF: verbal fluency; WAIS-R: Wechsler Adult Intelligence Scale—Revised.

**Table 4:** PEDro scale of the studies included.

Study	1	2	3	4	5	6	7	8	9	10	11	Total
Brüchle et al., 2021 [30]	1	0	0	1	0	0	1	0	1	1	1	5
Olson et al., 2017 [33]	1	1	0	1	0	0	0	0	0	1	1	4
Krogh et al., 2012 [34]	1	1	1	1	0	0	1	1	1	1	1	8
Buschert et al., 2019 [35]	1	1	0	1	0	0	0	0	1	1	1	5
Krogh et al., 2009 [36]	1	1	1	1	0	0	1	0	1	1	1	7
Zhang and Chen, 2019 [37]	1	0	0	1	0	0	1	0	1	1	1	5
Hoffman et al., 2008 [38]	0	1	0	1	0	0	1	0	1	1	1	6

PEDro: Physiotherapy Evidence Database scale; 1: eligibility criteria were specified (this item is not used to calculate PEDro score); 2: subjects were randomly allocated to groups; 3: allocation was concealed; 4: groups were similar at baseline regarding the most important prognostic indicators; 5: there was blinding of all subjects; 6: there was blinding of all therapists who administered the therapy; 7: there was blinding of all assessors who measured at least one key outcome; 8: measures of at least one key outcome were obtained from more than 85% of the subjects initially allocated to groups; 9: intention to treat analysis; 10: the results of between-group statistical comparisons are reported for at least one key outcome; 11: the study provides both point measures and measures of variability for at least one key outcome. Total: sum of items 2 to 11.

## Data Availability

Not applicable.

## Supplementary Materials

The following supporting information can be downloaded at: https://www.mdpi.com/article/10.3390/ijerph192215270/s1, Supplementary Data: search strategy and criteria inclusion.

## Appendix A

**Table A1:** Specific Search Strategy for Each Database.

Database	Search Strategy
EBSCO	TX (“executive functions” OR “cognitive function” OR cognition OR “inhibitory control” OR inhibition OR “working memory” OR “executive functioning” OR “cognitive flexibility”) AND TX (sport OR “modified sport” OR fitness OR exercise OR “physical activity” OR athletics OR “sport practice”) AND TX (depression OR “depressive disorder” OR “mood disorders” OR “major depressive disorder”)
PubMed	(((((((((“executive function”[Title/Abstract]) OR (“cognitive function”[Title/Abstract])) OR (cognition)) OR (“inhibitory control”[Title/Abstract])) OR (inhibition[Title/Abstract])) OR (“working memory”[Title/Abstract])) OR (“executive functioning”[Title/Abstract])) OR (“cognitive flexibility”[Title/Abstract])) AND (((((((sport[Title/Abstract]) OR (“modified sport”[Title/Abstract])) OR (fitness[Title/Abstract])) OR (exercise[Title/Abstract])) OR (“physical activity”[Title/Abstract])) OR (athletics[Title/Abstract])) OR (“sport practice”[Title/Abstract]))) AND ((((depression[Title/Abstract]) OR (“depressive disorder”[Title/Abstract])) OR (“mood disorders”[Title/Abstract])) OR (“major depressive disorder”[Title/Abstract]))
Scopus	((TITLE-ABS-KEY (depression)) OR (TITLE-ABS-KEY (“depressive disorder”)) OR (TITLE-ABS-KEY (“mood disorders”)) OR (TITLE-ABS-KEY (“major depressive disorder”))) AND ((TITLE-ABS-KEY (sport)) OR (TITLE-ABS-KEY (“modified sport”)) OR (TITLE-ABS-KEY (fitness)) OR (TITLE-ABS-KEY (exercise)) OR (TITLE-ABS-KEY (“physical activity”)) OR (TITLE-ABS-KEY (athletics)) OR (TITLE-ABS-KEY (“sport practice”))) AND ((TITLE-ABS-KEY (“executive function”)) OR (TITLE-ABS-KEY (“cognitive function”)) OR (TITLE-ABS-KEY (cognition)) OR (TITLE-ABS-KEY (“inhibitory control”)) OR (TITLE-ABS-KEY (inhibition)) OR (TITLE-ABS-KEY (“working memory”)) OR (TITLE-ABS-KEY (“executive functioning”)) OR (TITLE-ABS-KEY (“cognitive flexibility”)))
Web of Science	((TS = (“executive function” OR “cognitive function” OR cognition OR “inhibitory control” OR inhibition OR “working memory” OR “executive functioning” OR “cognitive flexibility”)) AND TS = (sport OR “modified sport” OR fitness OR exercise OR “physical activity” OR athletics OR “sport practice”)) AND TS = (depression OR “depressive disorder” OR “mood disorders” OR “major depressive disorder”)
